# Evaluation of p53 Gene Mutations in Oral Lichen Planus Lesions in a Population From Iran

**DOI:** 10.1002/cre2.70206

**Published:** 2025-08-28

**Authors:** Erfan Jokar, Mohamad Kazem Radaei, Vahid Poladvand, Abouzar Bagheri Haroni, Aetna Shiva, Maryam Seyedmajidi, Rouhallah Najjar Sadeghi

**Affiliations:** ^1^ Faculty of Medicine, Department of Clinical Biochemistry Mazandaran University of Medical Sciences Sari Iran; ^2^ Department of Nursing, School of Nursing and Midwifery Jiroft University of Medical Sciences Jiroft Iran; ^3^ Department of Clinical Biochemistry‐Biophysics and Genetics, Faculty of Medicine Mazandaran University of Medical Sciences Sari Iran; ^4^ Department of Oral and Maxillofacial Pathology, Faculty of Dentistry, Dental Research Center Mazandaran University of Medical Sciences Sari Iran; ^5^ Dental Materials Research Center, Health Research Institute Babol University of Medical Sciences Babol Iran; ^6^ Molecular and Cell Biology Research Center, Faculty of Medicine Mazandaran University of Medical Sciences Sari Iran

**Keywords:** DNA sequencing, oral lichen planus, p53, squamous cell carcinoma

## Abstract

**Objectives:**

Oral lichen planus (OLP) is a chronic inflammatory disorder of the oral mucosa, genetic and molecular alterations, including mutations in the p53 tumor suppressor gene, have been implicated in OLP pathogenesis. However, its molecular mechanisms are not clearly understood. This study investigates p53 gene mutations in OLP lesions.

**Material and Methods:**

This study analyzed 43 paraffin‐embedded tissue blocks from OLP patients. Diagnosis was confirmed by two pathologists. Genomic DNA was extracted using a commercial kit, with quality and quantity assessed by spectrophotometry and agarose gel electrophoresis. PCR‐sequencing was performed on exons 5–8 and part of the adjacent introns of the p53 gene. Statistical analysis was performed using SPSS version 20, with Fisher's exact test and *t*‐test applied to assess relationships between p53 mutations and clinical parameters; significance was set at *p* < 0.05.

**Results:**

The study included 43 OLP cases (mean age 51.7  ±  13.3 years; 65.1% female). Lesions were most frequently located on the buccal mucosa (65.1%), followed by the tongue (20.9%), gingiva (19.3%), and mandible (4.7%). DNA sequencing identified 13 nucleotide changes in the p53 gene in 9 samples (20.9%), distributed across exons 5–7 and intronic regions at codons 140, 171, 185, 213, and 246, as well as at IVS4: +8(C/G), IVS7: −11(A/C), and IVS4: −18(A/T). Mutations included equal proportions of missense and silent changes, as well as transitions and transversions. Adenine mutations were most common (53.8%), followed by cytosine mutations (30.8%). No statistically significant associations were found between p53 mutations and patient gender, age, or anatomical site of sampling.

**Conclusions:**

The study identified p53 gene mutations in 21% of oral lichen planus (OLP) cases, with no demographic or pathological correlations. While highlighting p53's complex role and potential as a biomarker, limited sample size necessitates larger, multi‐center studies to clarify genetic/environmental influences on OLP pathogenesis and mutation predisposition.

## 1 Introduction

Oral lichen planus (OLP) refers to a chronic inflammatory condition of the stratified squamous epithelia of the oral cavity that can affect the tongue, gingiva, floor of the mouth, lips, and palate (Louisy et al. 2024).

Meta‐analyses estimate the overall prevalence of OLP in the general population to be around 0.89%, clinic‐based studies indicate its prevalence to be highest in South America (3.18%) and the lowest in North America (0.11%) and India (0.49%), and Europe as 1.43% (Chiang et al. 2018; Vijayan 2022). Frequency of OLP in the Iranian population under the age of 18 years was discovered to be very low (Bakhtiari et al. 2017). While OLP is generally self‐limiting, the lesions can recur spontaneously and are a source of morbidity (Eisen et al. 2005).

The pathogenesis of OLP appears to be complex and multifactorial, involving genetic, immunological, environmental, and lifestyle factors (Gupta and Jawanda 2015). The exact underlying pathogenesis of OLP has not been clarified, but evidence suggests that autoimmunity and inflammation act as causative (Erichsen et al. 2018) or contributing factors, given the established association between chronic inflammation and various cancers (Afify et al. 2022). However, the transformation of normal epithelium is the result of various genetic mutations leading to deregulation of the cell cycle and alterations in the cells' differentiation (Matthews et al. 2022).

The p53 acts as a tumour suppressor and as a transcription factor, directly activates nearly 500 genes responsible for diverse cellular functions. In unstressed cells, p53 protein levels are low, but after some stress, such as DNA damages, nutrient and growth factors deprivation or oncogenic activity, p53 accumulates in the nucleus, where it regulates the cellular responses through induction of cell cycle arrest at the G1/S checkpoint, coordination of DNA damage repair systems and on the other hand, induces cell death by activating the intrinsic apoptotic pathway (Wang et al. 2022). This has become evident in the most human diseases; the normal function of wild‐type p53 is disrupted directly and indirectly by mutations and interactions with viral or other modified p53 binding proteins (Najjar and Sadeghi 2013). Accordingly, the outcome of p53 mutation and dysfunction is its inability to regulate DNA integrity and cell cycle arrest, and maybe to uncontrolled proliferation of damaged cells and tumorigenesis (Vaddavalli and Schumacher 2022).

Given the limited investigation into p53 gene mutations in oral lichen planus lesions specifically within the Iranian population, this study aims to address this gap. Understanding the prevalence and nature of p53 mutations in OLP could provide valuable insights into the molecular alterations associated with this condition in this demographic. Therefore, the present research focuses on characterizing p53 gene mutations in oral lichen planus among Iranian patients to contribute to the existing knowledge base and potentially inform future diagnostic or therapeutic strategies.

## 2 Materials and Methods

### 2.1 Patients and Tissue Samples

This study was conducted on 43 paraffin‐embedded blocks prepared from biopsies of patients diagnosed as OLP, including 15 samples of non‐erosive subtype and 28 samples of erosive subtype. These blocks were sourced from archives of the faculties of dentistry at Mazandaran University of Medical Sciences and Babol University of Medical Sciences, Iran, during 2017 to year 2020. The samples were examined by two pathologists to confirm the diagnosis. Ethical approval for this study was obtained from the Ethical Committee of Mazandaran University of Medical Sciences, Sari, Iran (IR.MAZUMS.REC.1398.5175).

### 2.2 Genomic DNA Extraction

To extract genomic DNA from paraffin‐embedded samples, three serial sections of 10‐μm thickness per sample were deparaffinized using xylene treatment. The extraction procedure followed the according to the instructions of the commercial kit from GeneAll Biotechnology Co. (GeneAll Exgene, FFPE tissue DNA, Korea). Subsequently, DNA was suspended in the buffer of choice, quantified and stored at −20°C until usage. The quality and quantity of the extracted DNA were evaluated by measuring the absorbance at 260 nm and calculation of A260/280 ratio using WPA Spectrophotometer (Biochrom WPA Biowave II UV/visible). In addition, the integrity of extracted DNA was checked using agarose gel electrophoresis.

### 2.3 PCR Amplification and Mutation Analysis Using Direct Sequencing

To find possible nucleotide alterations, polymerase chain reaction was carried out on extracted DNA and exons 5–8 of p53 gene were amplified and finally sequenced using the primers (NCBI Reference Sequence: NC_000017.11, region: 7669609‐767659) shown in Table 1.

**Table 1:** Primer sequences for exons 5–8 of the p53 gene.

		Sequence	Product length
Exon 5	F	5' CTGTTCACTTGTGCCCTGAC 3'	272 bp
	R	5' AACCAGCCCTGTCGTCTCT 3'	
Exon 6	F	5' CCTCTGATTCCTCACTGATTGC 3'	193 bp
	R	5' ACTGACAACCACCCTTAACC 3'	
Exon 7	F	5' GGTTGGCTCTGACTGTACCAC 3'	233 bp
	R	5' GTGATGAGAGGTGGATGGGTAG 3'	
Exon 8	F	5' AGGGTGGTTGGGAGTAGATGG 3'	260 bp
	R	5' ACCGCTTCTTGTCCTGCTTG 3'	

PCR was carried out in 25 µL of reaction containing 1× PCR buffer, 37 mM MgCl_2_, 10 pmol of each primer, 200 mM of each dNTP, and 0.5 U Taq polymerase. The PCR conditions for each region were as follows: initial cycle of 5 min at 94°C and then 30 cycles of 30 s at 94°C, annealing (30 s at 62.1°C for exon 5, 59.5°C for exon 6, 63°C for exons 7 and 6, 62.1°C for exon 8), 45 s at 72°C that was concluded by 10 min at 72°C. DNA sequencing was performed using ABI3130X Genetic Analyzer.

### 2.4 Statistical Analysis

The data was analyzed using SPSS version 20 statistical software and Fisher's exact test and *t*‐test was used to assess the relationship between mutations of the p53 gene and the clinicopathological parameters. Statistically significant in this study was a P value of less than 0.05.

## 3 Results

### 3.1 Patients' Findings

Forty‐three confirmed samples of OLP were included in the study, mean age of 51.7 ± 13.3 years, age range 17–70 years, 15 males (34.9%, mean age: 49.5 ± 15.1), 28 females (65.1%, mean age: 52.9 ± 12.3 years), and the female‐to‐male ratio was 1.87. The OLP lesions biopsied from buccal mucosa (65.1%) were the most commonly affected site, followed by tongue (20.9%), gingiva (9.3%), and mandible (4.7%). Size of greatest dimension of lesion was vary 0.4–2 cm with mean of 0.863 ± 0.29 cm (mean ± SD).

### 3.2 DNA Sequencing

**Figure 1:**
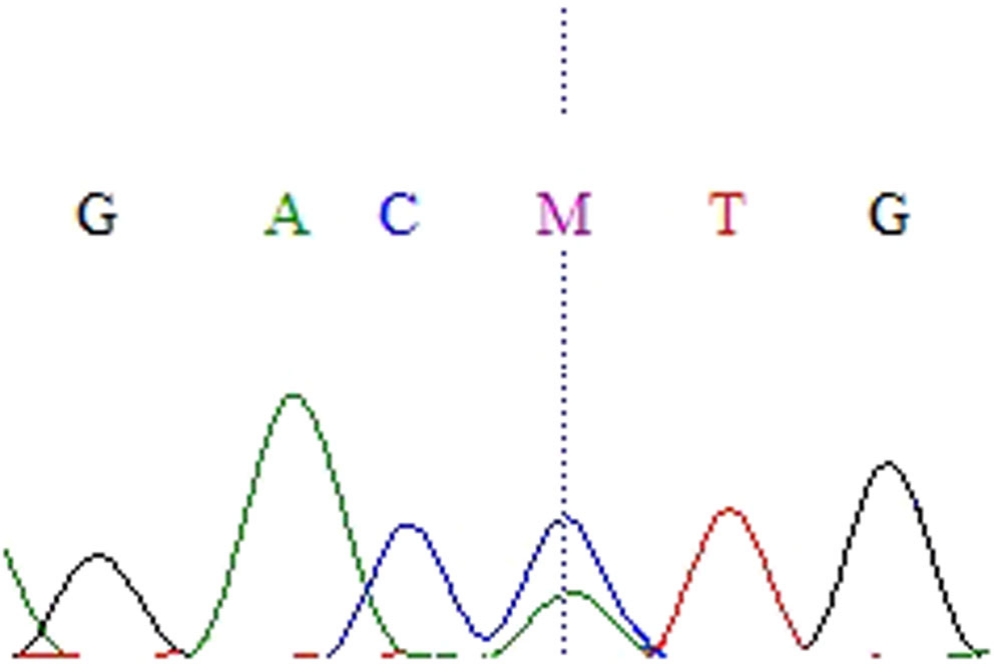
Cytosine to adenine base change in codon 140 of exon 5.

**Figure 2:**
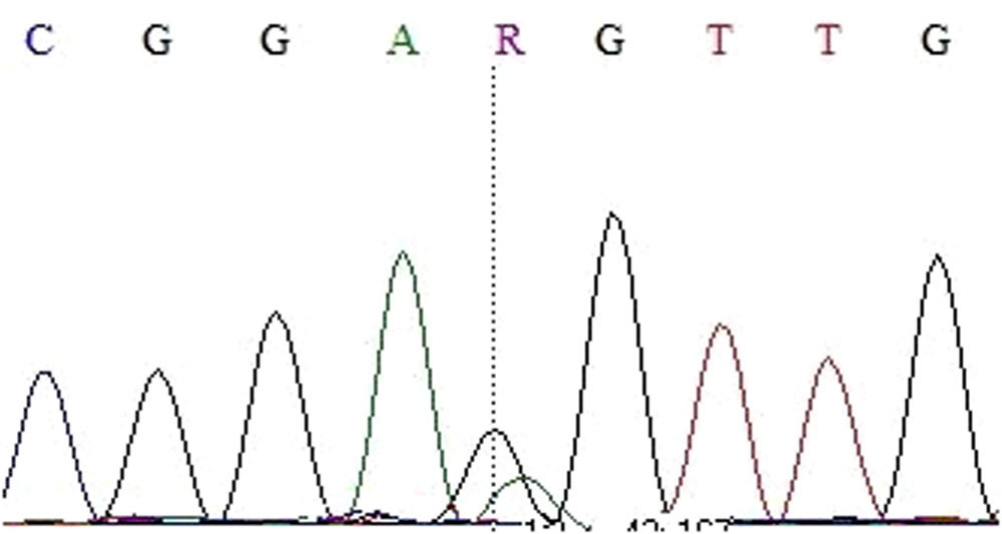
Guanine to adenine base change in codon 170 of exon 5.

**Figure 3:**
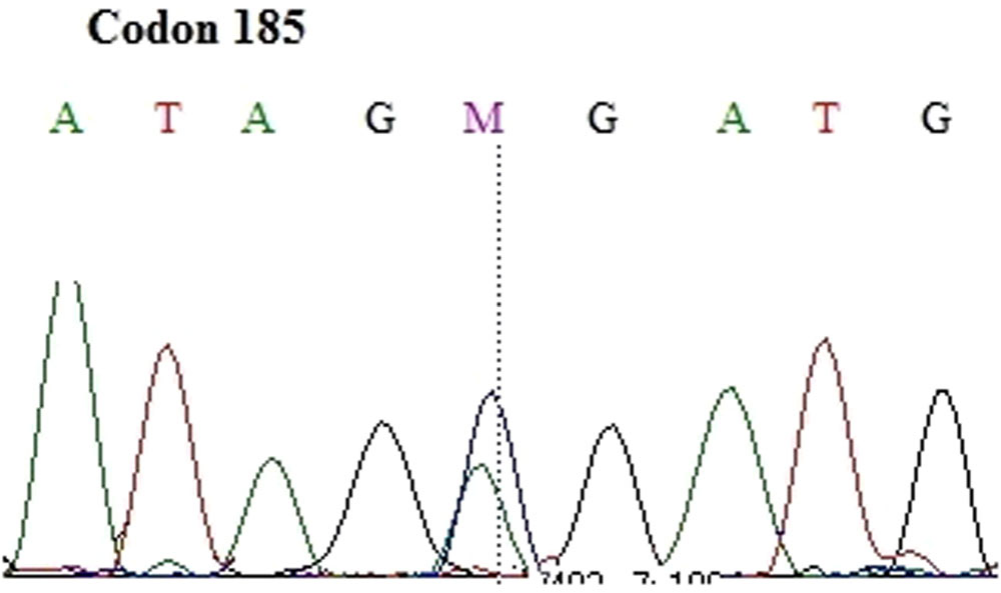
Cytosine to adenine base change in codon 185 of exon 5.

**Figure 4:**
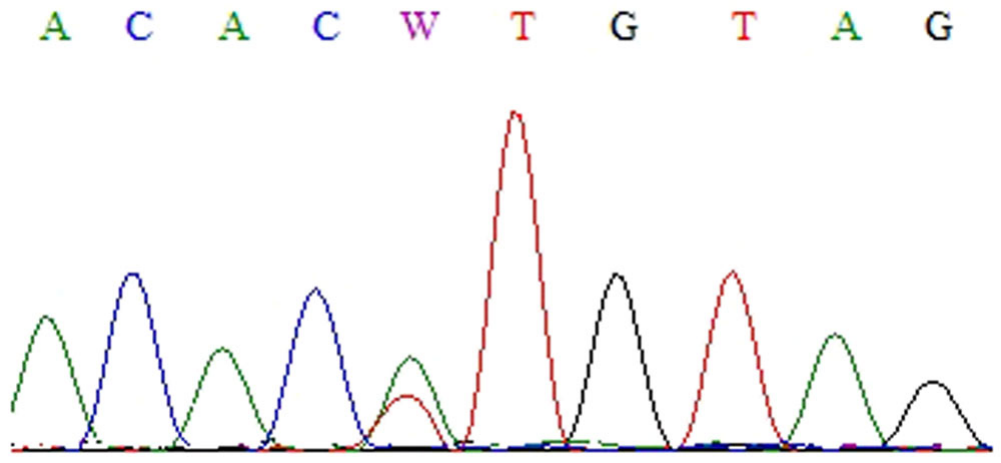
Change of adenine base to guanine base in codon 237 of exon 6.

**Figure 5:**
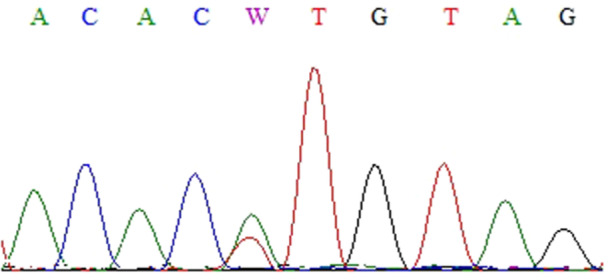
Nucleotide of thymine base to adenine base in codon 213 of exon 7. This exon was sequenced by reverse primer.

**Figure 6:**
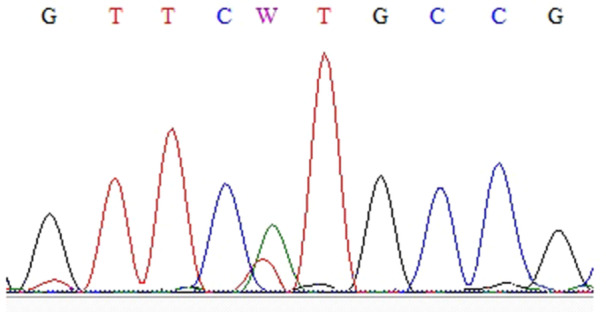
Nucleotide change of thymine base to adenine base in codon 246 of exon 7. This exon was sequenced by reverse primer.

Thirteen nucleotide changes (Figures 1, 2, 3, 4, 5, 6) were seen in 9 sample out of 43 (20.9%) as follow: three alterations in exon 5 (codon 140,171 and 185), three in exon 6 (codon 213), two in exon 7 (codon 237 and 246), one in IVS4: +8(C/G), two at IVS7:‐11(A/C) and also, two at IVS4‐18(A/T) (Table 2). Of the mutations within exons were as follows: 4 missense (50%), 4 (50%) silent, 4 (50%) transition, and 4 (50%) transversion.

**Table 2:** The frequency and type of mutations in each exon of p53.

	Exon 5	Exon 5	Exon 5	Exon 6	Exon 7			
Codon no.	140	185	171	213	237 and 246	IVS4‐18	IVS7: −11	IVS4: +8
Codon	ACC	AGC	GAG	CGA	ATG			
Changes	AAC	AGA	GAA	CGG	AAG			
Amino acid change	Thr > Asn	Ser > Arg	Glu > Glu	Arg > Arg	Met > Lys			
Effect	Missense	Missense	Silent	Silent	Missense			
Number	1	1	1	3	2			
Type	Trv.	Trv.	Trs.	Trs.	Trv.	Trv.	Trv.	Trv.
Nucleotide change	C > A		G > A	A > G	T > A	A > T	A > C	C > G
Number (%)	2 (15.38)		1 (7.7)	3 (23)	2 (15.38)	2 (15.38)	2 (15.38)	1 (7.7)

The frequency of adenine mutation (A > G, A > T, and A > C) was higher than other types of alterations (53.8% vs. 46.2%). Cytosine mutation (C > A, C > G and G > A) was the second type of alteration (30.8%), of which only one (7.7%) mutation (codon 185, C > A transversion) occurred at CpG dinucleotide (Table 2). The frequency of p53 gene mutation (Table 3) as not different statistically between male and female (Fisher's Exact Test *p* value > 0.05). The mean of age (Table 3) of the group with mutation and group without mutation was not different statistically (*t* test *p* value = 0.788). There was no statistical difference between the site of sampling (Table 3) and the mutation (Fisher's Exact *t* test *p* value = 0.3).

**Table 3:** The frequency of mutations in relation to gender, age, and sites of sampling.

	Gender, number (%)		Age		Site of sampling, number (%)			
Mutation	Male	Female	Mean ± SD	Range	Buccal	Gingiva	Mandible	Tongue
Yes	3 (20)	6 (21.5)	52.67 ± 10.9	33–70	4 (14.3)	1 (15)	1 (50)	3 (33.3)
No	12 (80)	22 (78.5)	51.47 ± 13.9	17–70	24 (85.7)	3 (75)	1 (50)	6 (66.7)

## 4 Discussion

Oral lichen planus is a T‐cell‐mediated disease of uncertain etiology that affects the oral mucosa (Elenbaas et al. 2022). According to some reports, some subtypes of OLP (atrophic and erosive subtypes) have a high risk of get involved in oral squamous cell carcinoma or cancer in other sites (González‐Moles et al. 2019). However, the exact molecular and pathological mechanism of OLP is not yet specified.

The function of p53 in maintaining genomic stability and integrity is well established (Janic et al. 2025). Mutations in p53 can lead to loss of its tumor suppressor activity, enabling cells with damaged DNA to survive and proliferate, contributing to increased malignant transformation risk (Zawacka‐Pankau 2022). Mutations of p53 have been identified in various diseases and cancers (D'Orazi 2023).

According to our findings, most of the patients were female and with a mean age at diagnosis of 51.7 years. These findings were in accordance with earlier work denoting that OLP usually affects adult older than 45 years with a mean age at diagnosis being 50–55 years and predominantly involve woman (Oliveira Alves et al. 2010). Similarly, the female‐to‐male ratio of 1.87 observed in this study reflects the higher prevalence of OLP in females, as previous reports that have reported the ratio as 1.4–1.8:1 (Chiang et al. 2018; Netha et al. 2022; Vijayan 2022).

Any site of the buccal cavity may be affected by OLP. However, in accordance with earlier reports, buccal and tongue are the common sites of involvement (Chiang et al. 2018; Vijayan 2022).

Based on our findings, 20.9% of OLP specimens had a mutation in p53 gene. However, to date, few mutational analyses have been conducted on p53 gene in OLP lesions, while they also provide inconsistent results. Ögmundsdottir et al. (2002) reported p53 gene mutation in 33% of OLP samples using a constant denaturation gel electrophoresis method, whereas Schifter et al. detected any cases with mutation at exons 5–9 of p53 gene using PCR‐SSCP (Schifter et al. 1998). On the other hand, the only previous report from Iran has focused on codon 72 alteration, showing in OLP the frequency of proline allele was significantly higher than that of arginine (Tabatabaei et al. 2018). Such disagreements can be attributed to methods of tissue sampling, DNA extraction, and possibly to the technique used to identify the mutation. In the present study, we used direct sequencing to identify probable alterations. The frequency of p53 mutations in OSCC varies from 21% to 35% and it was noted that in all of these reports, p53 protein expression was not associated with p53 mutation (Ögmundsdóttir et al. 2002; Ara et al. 2014).

A notable finding was that more than 50% of detected mutations were located within exons 5–7 of the p53 gene, with an equal distribution between missense and silent mutations. Missense mutations are particularly significant since they have the potential to alter the structure and function of the resultant protein. Silent mutations, while not altering protein sequence but may affect splicing elements (González‐Moles et al. 2019) or mRNA expression and stability (Chiang et al. 2018; D'Orazi 2023).

The predominance of exonic mutations emphasizes their potential role in disrupting the ability of p53 to regulate apoptosis and DNA repair pathways. Furthermore, mutations within the exons 5–7 of p53 are known hotspot sites in various cancers (Baugh et al. 2018), reinforcing their importance in the conditions such as OLP.

The higher frequency of adenine substitutions (53.8%) compared to other alterations is a striking observation, suggesting its specific susceptibility within the nucleotide sequence of p53 in the context of OLP lesions. Adenine mutations, particularly transitions (e.g., A > G) may arise from oxidative stress or exposure to environmental carcinogens (Vijayan 2022). This finding warrants further investigation into the molecular mechanisms driving adenine‐specific mutagenesis.

Interestingly, only one mutation occurred at a CpG dinucleotide, a known hotspot for methylation‐induced mutagenesis (Chiang et al. 2018). This contrasts with findings in other conditions where CpG sites are frequently mutated (Louisy et al. 2024). The low frequency of CpG mutations may reflect differences in epigenetic regulation or DNA repair mechanisms specific to OLP.

There was no statistically significant correlation between age or gender and p53 mutations in the current study. This is in accordance with previous reports suggesting that in OLP, p53 mutations are not influenced by demographic factors such as age or sex (Tabatabaei et al. 2018; Jayaraj et al. 2020) and implies that hormonal processes may not directly impact p53 mutational patterns. Despite the lack of statistical differences, it is worth noting that the mean age of patients with mutations was slightly higher than those without mutations. This observation could suggest a cumulative effect of environmental or cellular stresses over time, potentially contributing to genetic alterations in older individuals. However, further research with larger sample sizes is required to confirm this trend.

Any site of the buccal cavity may be affected by OLP. However, in conformity with earlier reports, we found that the buccal and tongue are the commonest sites of involvement (Chiang et al. 2018; Vijayan 2022). There was no association between the site of biopsy and mutation frequency. This suggests that p53 mutations are more likely be a generalized feature of OLP lesions rather than being site‐specific. However, it is worth noting that buccal mucosa lesions are often subjected to chronic irritation from mechanical forces or chemical and environmental exposures, which can indirectly influence mutational events.

Despite the rare mutational studies, many studies have been conducted for protein expression analysis by the IHC technique. Previous studies have shown increased p53 expression in OLP. In agreement, most authors found that p53 protein expression is a mostly frequent events in OLP (Chiang et al. 2018; D'Orazi 2023; Keim‐Del Pino et al. 2024). It is suggesting that increased p53 protein expression, even in small percentages of cells, might indicate mutant forms of the protein that are more stable and potentially carcinogenic. However, on some occasions, p53 protein overexpression was not consistent with the significantly lower frequency of the observed mutations (Ögmundsdóttir et al. 2002; Ara et al. 2014). Therefore, it has been suggested that enhanced expression of p53 protein might be secondary to cellular stress due to inflammatory conditions in OLP (Ögmundsdóttir et al. 2002) or may present a physiological response to the hyperproliferative state of OLP (Schifter et al. 1998) and also, is also induced by wild‐type p53, which tries to arrest the cell cycle in response to DNA damage (Engeland 2022). Therefore, there is not necessarily any causal relationship between IHC results and mutations present in the p53 gene, and this emphasizes the need for further studies exploring how specific mutations influence p53 functionality in OLP lesions.

## 5 Conclusion

This study investigated the molecular characteristics and mutational profile of the p53 gene in OLP lesions, revealing diverse mutations in about 21% of cases without demographic and pathologic correlation. These highlighted the complexity of p53 involvement in OLP and its potential AC as a universal biomarker for OLP pathogenesis. Despite these findings, the study's limited sample size, necessitates further larger, multicentral and longitudinal research to clarify its exact role in OLP development and pathogenesis and also to determine whether demographic and environmental or genetic factors predispose some people to these alterations and ultimately to OLP.

## Author Contributions

**Erfan Jokar:** investigation, visualization, writing – original draft preparation. **Mohamad Kazem Radaei:** investigation, visualization, writing – original draft preparation. **Vahid Poladvand:** provision of study materials, original draft preparation. **Abouzar Bagheri Haroni:** methodology, review and editing. **Aetna Shiva:** provision of study materials, validation, and original draft preparation. **Maryam Seyedmajidi:** provision of study materials, validation, and original draft preparation. **Rouhallah Najjar Sadeghi:** conceptualization, methodology, supervision, review and editing.

## Conflicts of Interest

The authors declare no conflicts of interest.

## Data Availability

Data sharing is not applicable to this article as no new data were created or analyzed in this study.
